# Marine-Derived Compounds and Prospects for Their Antifungal Application

**DOI:** 10.3390/molecules25245856

**Published:** 2020-12-11

**Authors:** Joana Cardoso, Darlan Gonçalves Nakayama, Emília Sousa, Eugénia Pinto

**Affiliations:** 1 Laboratório de Microbiologia, Departamento de Ciências Biológicas, Faculdade de Farmácia, Universidade do Porto, Rua de Jorge Viterbo Ferreira 228, 4050-313 Porto, Portugal; joanina_cardoso@hotmail.com (J.C.); darlan_nakayama@hotmail.com (D.G.N.); 2 Laboratório de Química Orgânica e Farmacêutica, Departamento de Ciências Químicas, Faculdade de Farmácia, Universidade do Porto, Rua de Jorge Viterbo Ferreira 228, 4050-313 Porto, Portugal; 3 CIIMAR—Interdisciplinary Centre of Marine and Environmental Research, Terminal de Cruzeiros do Porto de Leixões, 4450-208 Matosinhos, Portugal

**Keywords:** fungal infections, antifungal resistance, marine natural products, new antifungal approaches

## Abstract

The introduction of antifungals in clinical practice has an enormous impact on the provision of medical care, increasing the expectancy and quality of life mainly of immunocompromised patients. However, the emergence of pathogenic fungi that are resistant and multi-resistant to the existing antifungal therapy has culminated in fungal infections that are almost impossible to treat. Therefore, there is an urgent need to discover new strategies. The marine environment has proven to be a promising rich resource for the discovery and development of new antifungal compounds. Thus, this review summarizes more than one hundred marine natural products, or their derivatives, which are categorized according to their sources—sponges, bacteria, fungi, and sea cucumbers—as potential candidates as antifungal agents. In addition, this review focus on recent developments using marine antifungal compounds as new and effective approaches for the treatment of infections caused by resistant and multi-resistant pathogenic fungi and/or biofilm formation; other perspectives on antifungal marine products highlight new mechanisms of action, the combination of antifungal and non-antifungal agents, and the use of nanoparticles and anti-virulence therapy.

## 1. Introduction

Fungi are a ubiquitous group of organisms occurring in a wide variety of habitats and ecological niches. They can be found in various environments: tropical forests, grasslands, deserts, coastal areas, ocean deeps, seas, freshwaters, and polar regions [1,2,3,4,5]. They are essential for many ecosystems, being important decomposers that contribute to the degradation of organic matter, with a fundamental role in ecological and biogeochemical processes. It is evident that fungi have a great role for human society. Fungi are used in medicine industry to obtain important drugs such as antibiotics, immunosuppressant, vitamins, steroids, and alkaloids. Genetically modified fungi can also be used as bio-factories for the production of numerous enzymes with the most diverse biotechnological applications [6,7,8]. However, some fungi can harm human life, causing many infections that can be classified into superficial/subcutaneous (dermatophytosis, pityriasis versicolor, and sporotrichosis) and systemic mycoses (candidiasis, aspergillosis, and cryptococcosis). These can target any type of tissue or organ and causing, for example, bone, lung, and neurological injuries [9].

The discovery of antifungal agents has allowed significant advances in medical care enabling the control and cure of fungal infections, altering their natural evolution. However, the development of resistance to antifungals and the fungal infections growth in the immunocompromised population have contributed, in recent decades, to the increase in the appearance and severity of these infections, resulting in an important cause of hospital death [10]. Systemic fungal infections are particularly common in immunocompromised patients, which accommodate not only those infected with the human immunodeficiency virus (HIV) but also transplanted and cancer patients [11,12]. Although most infections appear superficially, i.e., on the skin, hair, nails or mucous membranes, some fungal species, under conditions of the fragility of the immune system, are capable of causing systemic infections that can lead to death [11]. Invasive fungal infections are considered a serious threat to public health, being responsible for about 1.5 million deaths per year with a mortality rate until 90%, which is associated with species belonging to *Candida*, *Aspergillus*, *Cryptococcus*, *Pneumocystis*, *Mucor*, and *Rhizopus* genera [10,11,12,13]. In the genus *Candida, C. albicans* is the main cause of fungal infections, accounting for between 50 and 70% of cases. However, recent epidemiological data have revealed an increase in species resistant to most or all classes of antifungals called *Candida* non-*albicans*. These species have emerged from primary (intrinsic) resistance mechanisms or the development of secondary (acquired) resistance through the inappropriate use of antifungal agents [14,15,16]. Regarding *Aspergillus* spp., a change in epidemiology has also been observed in the last two decades resulting from the selective pressure exerted by the indiscriminate use of antifungals and the increase in immunocompromised patients, with intensification in the identification of emerging *Aspergillus* non-*fumigatus* and cryptic species resistant to various classes of antifungals, particularly to azoles [16,17]. Due to the significant increase in the number of resistant and multi-resistant strains, particularly in *Candida* spp. and *Aspergillus* spp., there is an urgent clinical need to discover and develop new antifungal agents to enable the renewal of the therapeutic arsenal and thus control the appearance of new resistances [11,12,18].

**Figure 1:**
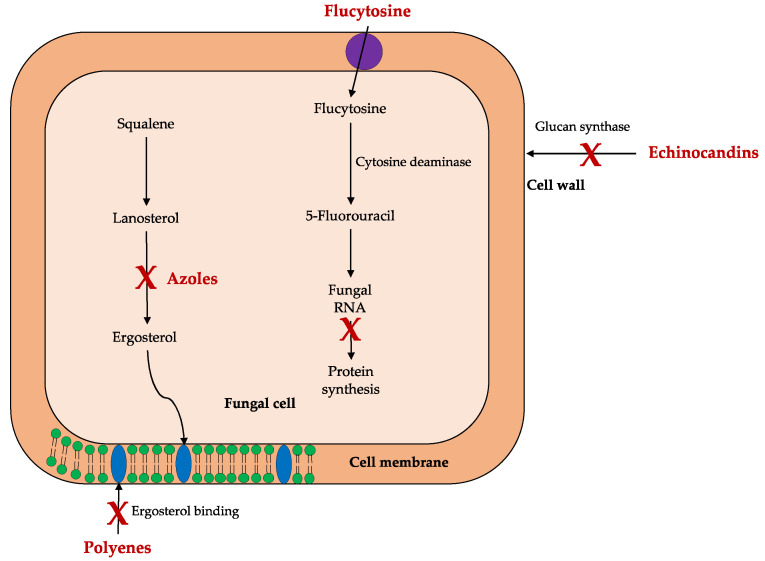
Mechanism of action of antifungal classes.

In the treatment of systemic mycoses, antifungals can be limited to four classes—polyenes, azoles, flucytosine, and echinocandins—in which they are distinguished by the mode of action (Figure 1), bioavailability, formulation, pharmacological interactions, and adverse effects [11,19].

**Figure 2:**
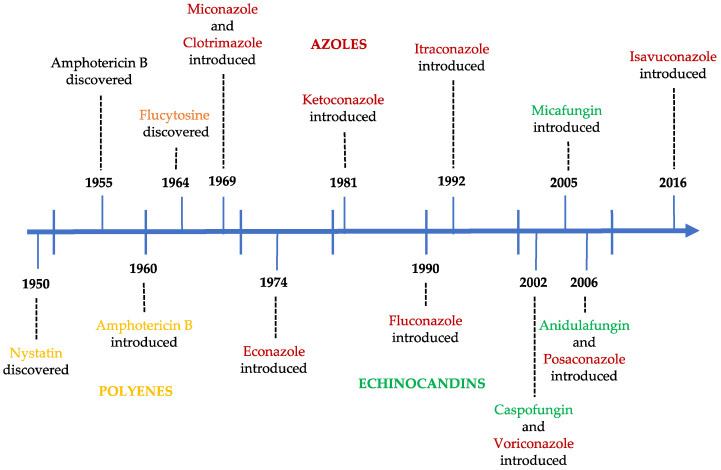
Historical evolution of antifungal agents.

Figure 2 represents the year of discovery and/or introduction to the market of the different antifungals. At the moment, no new class has been approved by the European Medicine Agency (EMA) or the Food and Drug Administration (FDA), which poses a problem, because the current antifungals agents are not sufficiently active and safe, and they exhibit some toxicity and undesirable adverse effects [10,13]. The appearance of a second generation of azoles and, more recently, of echinocandins are two important milestones in the efficacy of antifungal therapy. The discovery of new antifungal agents has mainly been associated to the screening of large libraries of small molecules of natural, synthetic, or semi-synthetic products [10,11,13].

The development of antifungals agents is a very challenging strategy, since there are physiological similarities between fungal cells and human cells, being responsible for toxicity. The disinvestment of the pharmaceutical industry, which focuses mostly on other more profitable areas, and the lack of economic incentives are other factors that have led to a shortage of new antifungal agents. Therefore, the development of new antifungals has been gradual compared to the development of new antibacterial agents [10,11,13,20,21]. A new antifungal agent should have few pharmacological interactions, adequate pharmacokinetic and pharmacodynamic properties, fungicidal nature instead of fungistatic action, and a broad spectrum of activity against resistant and multi-resistant pathogenic fungi, preferably having a new mechanism of action that could reduce or prevent resistance and toxicity in the host. To discover such an agent, it is necessary to identify new fungal-specific targets that are essential for their fungal growth, and that the antifungal could be capable of eliminating fungal cells and not human cells [10,11,12]. Nevertheless, not all molecules that exhibit antifungal activity can be optimized for the development of new antifungal agents due to undesirable characteristics [12].

Marine biodiversity corresponds to the variety of organisms found in marine and oceanic ecosystems, comprising more than 250,000 species described and infinity of other species to be discovered yet. These organisms can live in environments of extreme variation in pressure, luminosity, salinity, and temperature, being able to produce secondary metabolites that help in their survival. These unique metabolites can constitute a vast source of beneficial bioactive products in human health. In the last five decades, more than 13,000 bioactive marine natural products have been identified presenting various biological activities such as antibacterial, antidiabetic, antifungal, anti-inflammatory, and anticancer [22,23]. The prospecting for new antifungals of marine origin, different from those commercially available, with more effective pharmacokinetic and pharmacodynamic properties is already a reality, which will help in the treatment of human fungal diseases, mainly those caused by resistant and multi-resistant fungi.

From this point of view, this review will highlight new natural products isolated from diverse marine organisms and their antifungal activities, thus providing an overview of marine compounds with in vitro potential as antifungal agents. Recent and documented perspectives for potential applications of marine antifungal compounds will also be highlighted, including a new alternative target with a new mechanism of action, the interaction between antifungal and non-antifungal agents, and the use of nanoparticles and anti-virulence therapy, as a way to increase the activity of antifungals currently available, achieve the synergistic effect, and/or inhibit the formation of virulence factors, specifically of biofilms. These new strategies could enrich the pipeline of the treatment of fungal human infections and, consequently, resolve the limited number of therapeutic options related to the lack of new antifungals agents, obtain more diverse potential targets for action and solutions to combat the resistance acquired by many strains of pathogenic fungi.

## 2. Marine Natural Products as New Antifungal Candidates

Up to now, several compounds derived from secondary metabolites and isolated from marine organisms exhibited antifungal activity. Of all the marine organisms investigated, sponges are recognized as the main prolific source of secondary metabolites with antifungal properties, followed by bacteria and fungi. The chemical diversity of the bioactive compounds produced by these microorganisms is remarkable and, therefore, it can be considered essential for the discovery and development of new agents for the treatment and prevention of various fungal infections [24,25,26,27,28]. Sea cucumbers are also considered a rich source of bioactive and distinct secondary metabolites, of which triterpene glycosides (saponins) are evident. Since they have unique characteristics, involving great chemical diversity, low toxicity, high efficiency with few adverse effects, and a wide spectrum of antifungal activity, these natural products have gained a lot of attention, making them more favorable as leading compounds for the discovery of other new agents [29]. Thus, in the following sections, compounds with promising antifungal activity produced by marine microorganisms involving sponges, bacteria, fungi, and sea cucumbers will be highlighted.

### 2.1. Sponge-Derived Compounds

**Figure 3:**
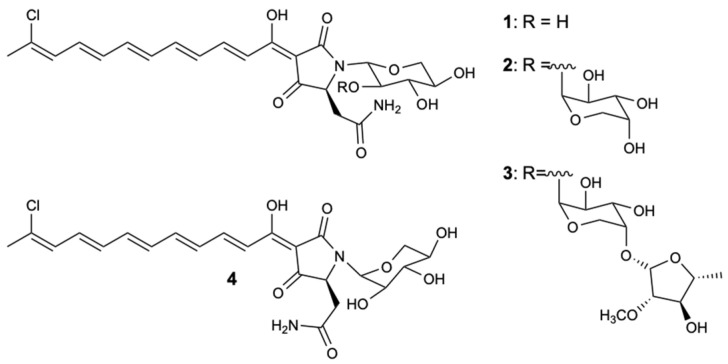
Structure of aurantosides G–J (**1**–**4**).

Aurantosides belong to a class of molecules produced by sponges of the genera *Theonella*, *Homophymia,* and *Siliquariaspongia*, which chemically present a tetramate ring with a mono- or dichlorinated long conjugated polyene chain and a *N*-glycosidic portion of one to three monosaccharides [30,31]. The study of the sponge *Theonella swinhoei* resulted in the extraction of three known aurantosides G–I (**1**–**3**) and a new aurantoside J (**4**) (Figure 3), which were tested for antifungal activity against *C. albicans*, *C. glabrata*, *C. parapsilosis*, *C. tropicalis*, and *Fusarium solani*. Compound **1** demonstrated moderate inhibition against all strains with MIC_90_ = 4–16 mg/L, and compound **3** was the only one that exhibited potent action with MIC_90_ values of 0.125–0.5 and 2 mg/L for *Candida* spp. and *F. solani*, respectively. Given the results obtained from compound **3**, it was found that the three sugar chains attached to the tetramate ring by the nitrogen atom, as well as the C18 polyene chain, were important descriptors for inhibiting the growth of the five fungal strains [30].

**Figure 4:**
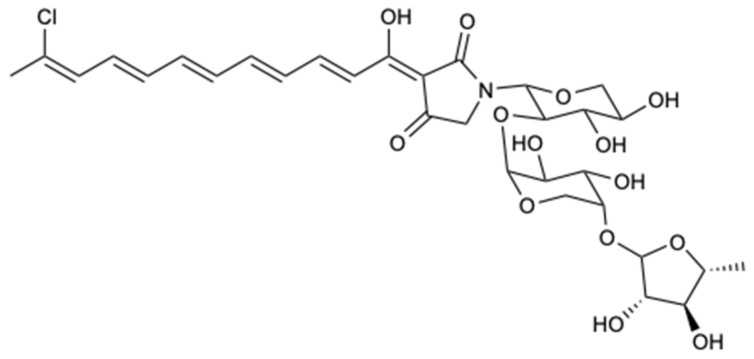
Structure of aurantoside K (**5**).

Although there are many reports of aurantosides discovered on sponges of the genus *Theonella*, a new derivative of tetramic acid glycoside, called aurantoside K (**5**) (Figure 4), was isolated from a marine sponge of the *Meophlus* sp. This compound showed strong antifungal inhibition under a broad spectrum of pathogenic fungi: Amphotericin B (AmB)-resistant *C. albicans* and wild-type *C. albicans* with minimum inhibitory concentration (MIC) values of 31.25 and 1.95 mg/L, respectively, and *C. neoformans*, *A. niger*, *Rhizopus sporangia*, *Penicillium* sp., and *Sordaria* sp. with zones of inhibition of 14, 28, 21, 31, and 29 mm, respectively at 100 μg/disc [31].

**Figure 5:**
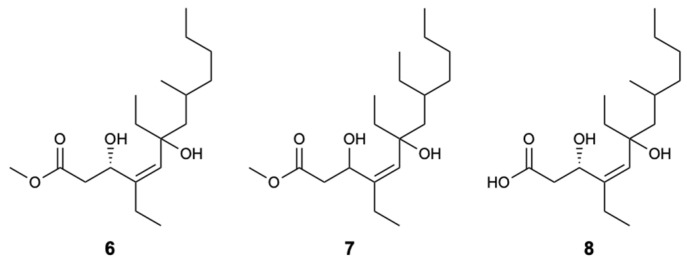
Structures of woodylides A–C (**6**–**8**).

Three new linear polyketides called woodylides A–C (**6**–**8**) (Figure 5), collected on the Xisha islands in the China Sea from the sponge *Plakortis simplex*, were evaluated for their antifungal activity against *C. albicans*, *C. neoformans*, *Nannizzia gypsea* (formerly *Microsporum gypseum*), and *Trichophyton rubrum*, with AmB and fluconazole as positive controls. Compounds **6** and **8** exhibited moderate action with IC_50_ values of 3.67 and 10.85 mg/L against *C. neoformans*, respectively, unlike compound **7**, which was not active. For the remaining fungi, compounds **6** and **8** showed weak activity and due to the lack of compound **7**, it was not possible to test their antifungal inhibition [32].

**Figure 6:**
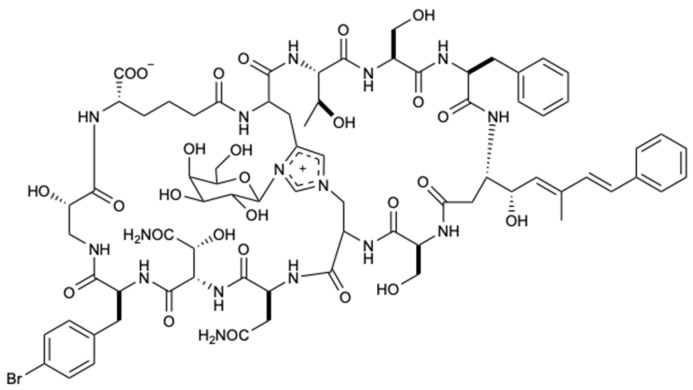
Structure of theonellamide G (**9**).

The genus *Theonella* is associated with biologically active peptides with immense structural diversity. In this way, a polar active portion of the organic extract of the sponge *Theonella swinhoei* was studied, which provided the isolation of theonellamide G (**9**) (Figure 6). This new bicyclic glycopeptide showed potent antifungal activity against wild-type *C. albicans* and AmB-resistant *C. albicans* with IC_50_ values of 4.49 and 2.0 μM, respectively. For wild-type, AmB was used as a positive control with an IC_50_ = 1.48 μM [33].

**Figure 7:**
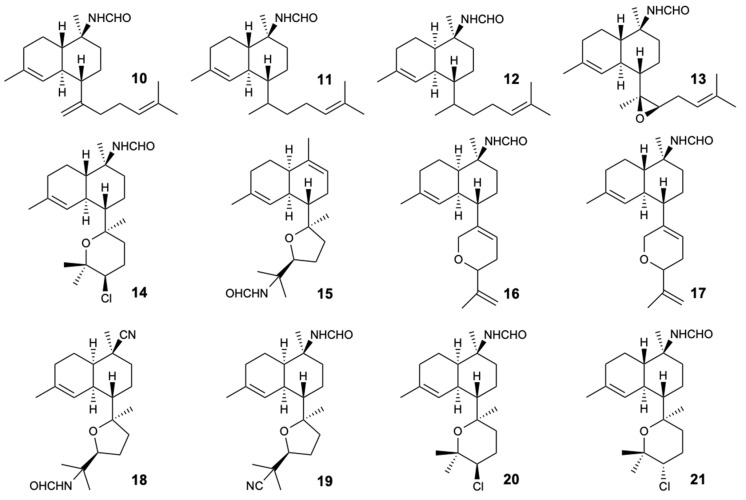
Structures of compounds **10**–**21**.

Seven new formamido-diterpenes, cavernenes A–D (**10**–**13**), kalihinenes E,F (**14**,**15**), and kalihipyran C (**16**), along with five known compounds, kalihipyran A (**17**), 15-formamidokalihinene (**18**), 10-formamido-kalihinene (**19**), and kalihinenes X,Y (**20**,**21**) (Figure 7), were isolated from the sponge *Acanthella cavernosa*. All compounds were tested for antifungal activity against *C. albicans*, *C. glabrata*, *C. parapsilosis*, *A. fumigatus*, *C. neoformans*, *N. gypsea*, and *T. rubrum*. Ketoconazole was used as a positive control with MIC ≤ 0.25 mg/L for all the tested strains. Compound **18** inhibited the growth of *N. gypsea* and *T. rubrum* with MIC values of 8 and 32 mg/L, respectively, and compound **19** showed action against *C. albicans*, *C. neoformans*, *N. gypsea*, and *T. rubrum* at concentrations of 8, 8, 8, and 4 mg/L, respectively. Given the results obtained, it was found that the isonitrile group is relevant for antifungal activity [34].

**Figure 8:**
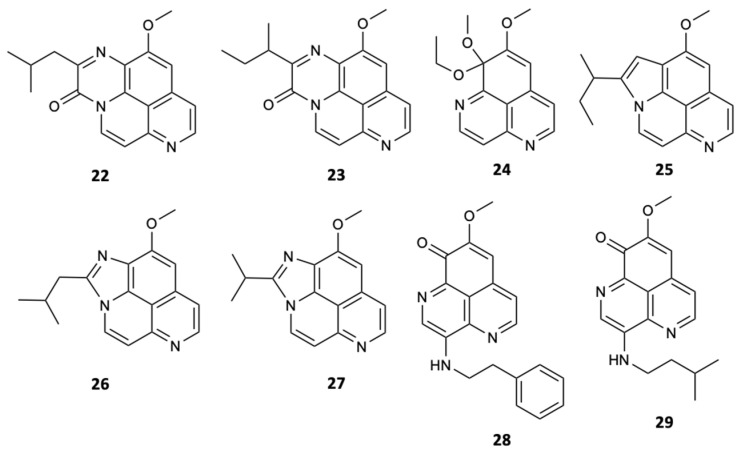
Structures of aaptamines (**22**–**29**).

Since 1982, with the discovery of the first aaptamine from the marine sponge *Aaptos aaptos*, several derivatives have been isolated from sponges belonging to the genera *Suberites*, *Luffariella*, *Hymeniacidon*, *Suberea*, and *Xestospongia*. Nevertheless, the genus *Aaptos* remains the main rich source of aaptamine alkaloids as bioactive secondary metabolites. Thus, numerous aaptamine derivatives with five new substances (**22**–**26**) and three known substances (**27**–**29**) (Figure 8) were extracted from the marine sponge *Aaptos aaptos*. All compounds, except **22** and **23** due to a lack of quantity, were tested for antifungal inhibition of *C. albicans*, *C. glabrata*, *C. parapsilosis*, *C. neoformans*, *N. gypsea*, and *T. rubrum*, in which fluconazole was used as a positive control. Compound **28** inhibited the growth of all studied fungi, except *C. glabrata*, at concentrations of 32, 64, 32, 16, and 4 mg/L, respectively. Compound **29** showed a strong action against *C. neoformans*, *N. gypsea*, and *T. rubrum* with MIC values of 64, 32, and 8 mg/L, respectively. The remaining compounds showed weak antifungal activity considering the tested fungi (MIC > 64 mg/L) [35].

**Figure 9:**
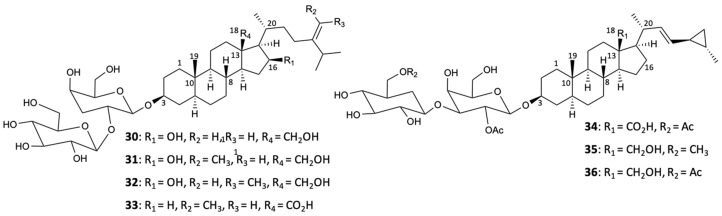
Structures of poecillastrosides A–G (**30**–**36**).

Until 2017, about 70 saponins were isolated from sponges, in which some of them, when submitted to different bioassays, revealed biological activities of great interest. The first study of the sponge *Poecillastra compressa*, by Bowebank in 1866, allowed finding seven new compounds, poecillastrosides A–G (**30**–**36**) (Figure 9). Later, these steroidal glycosides were tested for their ability to inhibit the growth of the fungus *A. fumigatus*, in which only compounds **33** and **34** exhibited activity with MIC_90_ values of 6 and 24 mg/L, respectively, concluding that the carboxylic acid present in C-18 plays an essential role in antifungal action [36].

**Figure 10:**
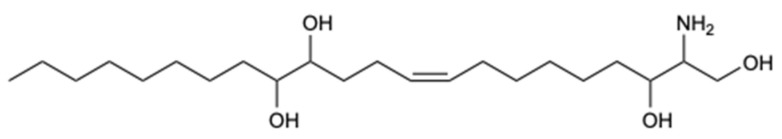
Structure of haliscosamine (**37**).

Preliminary studies with hydroalcoholic and organic extracts of the marine sponges *Haliclona viscosa* and *Cinchyrella tarentina* show potential antifungal activity against five phytopathogenic fungi: *F. oxysporum* f. sp. *melonis*, *F. oxysporum* f. sp. *radicis-lycopersici*, *F. oxysporum* f. sp. *ciceris*, *Botrytis cinerea*, and *Penicillium digitatum* [37]. Subsequently, a new derivative of sphingosine, called haliscosamine (**37**) (Figure 10), was isolated from *Haliclona viscosa* and showed significant in vitro activity against *C. albicans* (MIC_90_ = 0.4–0.8 mg/L), *C. tropicalis* (MIC_90_ = 0.4–0.8 mg/L), and *C. neoformans* (MIC_90_ = 0.2–0.4 mg/L) [38].

**Figure 11:**
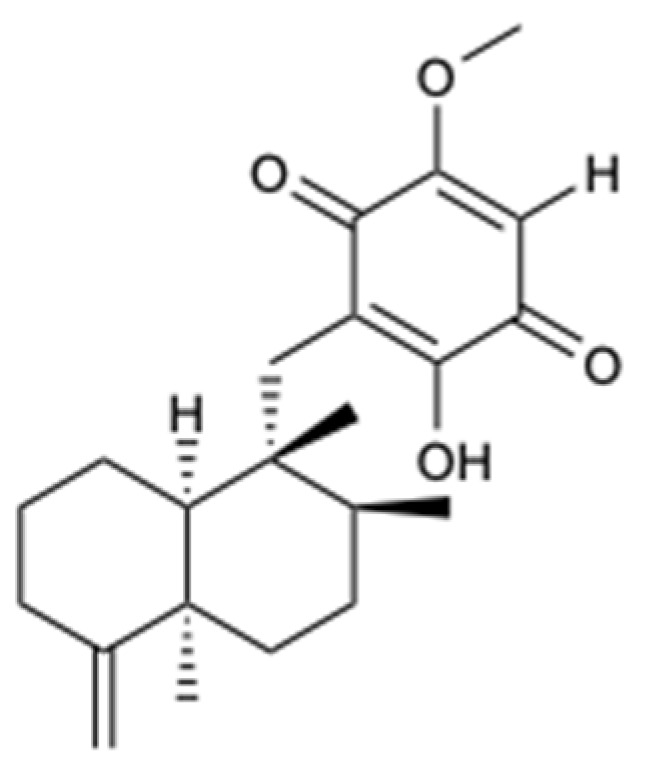
Structure of epi-ilimaquinone (**38**).

A sesquiterpenoid quinone, epi-ilimaquinone (**38**) (Figure 11), was isolated from the Fijian marine sponge, *Hippospongia* sp. This compound exhibited antifungal activity against AmB-resistant *C. albicans* (MIC = 125 mg/L), but it had no appreciable effect against *C. albicans*, *C. neoformans*, *A. niger*, *Penicillium* spp., *Rhizopus sporangia*, and *Sordaria* spp. [39].

### 2.2. Bacteria-Derived Compounds

**Figure 12:**
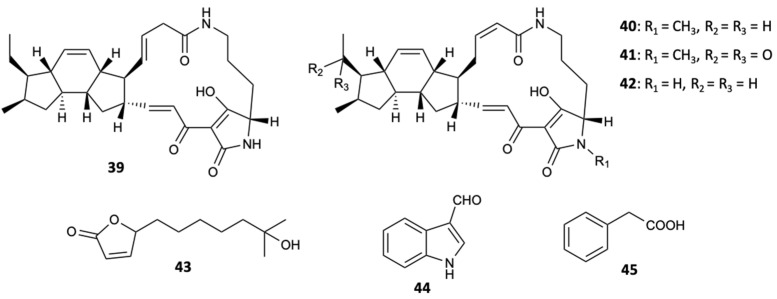
Structures of compounds **39**–**45**.

Bioassay guided fractionation from culture broths of the marine bacterial strain closely related to *Streptomyces zhaozhouensis* CA-185989 resulted in the extraction of three new polycyclic tetramic acid macrolactams, isoikarugamycin (**39**), 28-*N*-methylikaguramycin (**40**), and 30-oxo-28-*N*-methylikaguramycin (**41**), and four known molecules, ikarugamycin (**42**), MKN-003B (**43**), 1*H*-indole-3-carboxaldehyde (**44**), and phenylethanoic acid (**45**) (Figure 12). Compounds **39**, **40**, and **42** showed potent antifungal activity against *A. fumigatus*, with MIC values in the range 4–8 mg/L, and against *C. albicans*, with MIC = 2–4 mg/L for compound **39** and MIC = 4 mg/L for compounds **40** and **42**. The remaining compounds were not active when tested at a concentration of 64 mg/L. With the MIC values obtained for the compounds **39**, **40**, and **42**, it was found that the ethyl group plays an essential role in the antifungal action in the family of polycyclic tetramate macrolactams, since the existence of a carbonyl group in the ethyl side chain at C-16 is associated with an increase in the MIC, even though the *N*-methylation of the nitrogen atom of the tetramic acid portion does not affect the activity [40].

**Figure 13:**
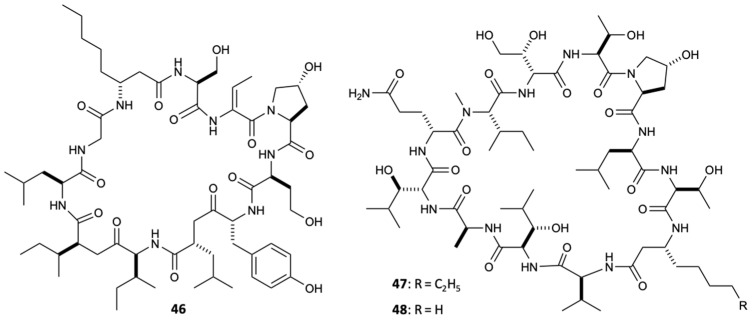
Structures of compounds **46**–**48**.

Filamentous marine cyanobacteria are one of the sources of bioactive secondary metabolites that naturally gain a lot of popularity in the study of marine natural products. Currently, there are numerous compounds from strains of cyanobacteria, mainly from the genera *Lyngbya*, *Oscillatoria*, and *Symploca* [41]. Lobocyclamides A–C (**46**–**48**) (Figure 13) are peptides isolated from *Lyngbya confervoides* that revealed significant activity against *C. glabrata* and *C. albicans*, in which the compounds **46** and **47** showed MIC values of 91 and 30–100 mg/L, respectively. The existence of mixtures consisting of compounds **46** and **47** (1:1) allowed investigating an important synergistic effect with great antifungal activity (MIC = 10–30 mg/L) compared to any of the pure isolated compounds [42].

**Figure 14:**
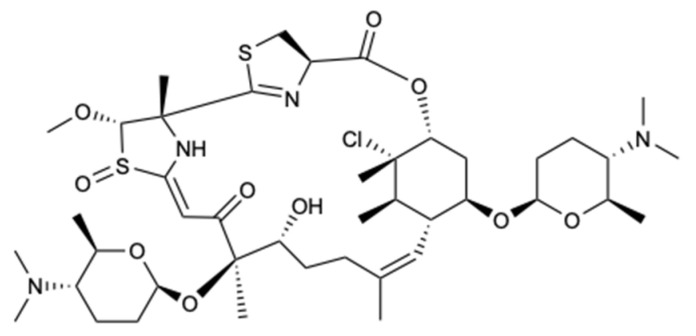
Structure of forazoline A (**49**).

A new polyketide compound, called forazoline A (**49**) (Figure 14), was isolated from *Actinomadura* spp., which was cultivated from the tunicate *Ecteinascidia turbinata*. Forazoline A exhibited potential antifungal effects in vitro (MIC < 16 mg/L) and in vivo against *C. albicans*. Furthermore, an in vitro combination treatment with forazoline A and AmB exhibited a synergistic antifungal effect, suggesting a parallel or complementary mechanism of action between these compounds [43].

### 2.3. Fungi-Derived Compounds

**Figure 15:**
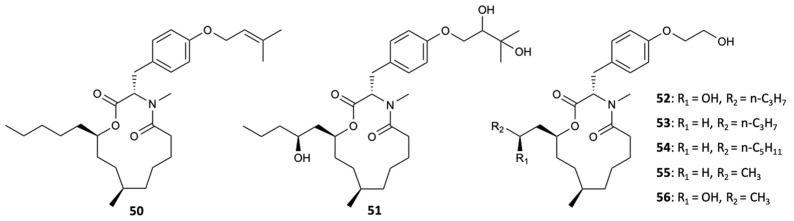
Structures of 13-membered macrolides (**50**–**56**).

Macrolides are a class of polyketides often discovered in organisms of marine origin with an important antifungal role [44]. In the 13-membered macrolides, two new compounds, melearoride A,B (**50**,**51**), and five known compounds, PF1163A (**52**), B (**53**), D (**54**), H (**55**), and F (**56**) (Figure 15), were isolated from a strain of a marine-derived fungus, *Penicillium meleagrinum* var*. viridiflavum*. The evaluation of antifungal activity against *C. albicans* demonstrated the inhibition of fungal growth for **52**, **53**, **55**, and **56** at concentrations of 1, 2, 16, and 8 mg/L, respectively, while for **50**, **51**, and **54**, no activity at a concentration of 32 mg/L was observed. It was also found that the hydroxyethyl group was essential for the activity of **52** and **53**, since they presented activity against *C. albicans*, unlike **50** and **51**, which showed weak antifungal action. Since the fungus understudy is resistant to the azole class, tests were carried out with fluconazole, in which the results showed that all compounds, including those that did not show activity, exhibited a synergistic effect with this commercialized antifungal [45].

**Figure 16:**
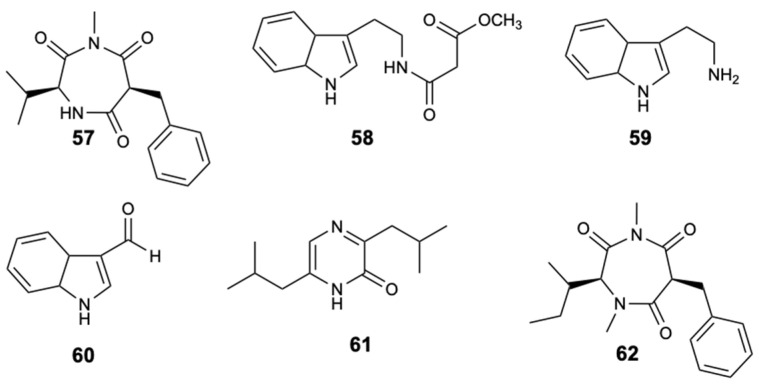
Structures of compounds **57**–**62**.

The chemical study of the tunicate-derived fungus *Penicillium* spp. CYE-87 resulted in the extraction of a new compound with the 1,4-diazepane skeleton, terretrione D (**57**), together with the five known ones, methyl-2-([2-(1*H*-indol-3-yl)ethyl]carbamoyl)acetate (**58**), tryptamine (**59**), indole-3-carbaldehyde (**60**), 3,6-diisobutylpyrazin-2(1*H*)-one (**61**), and terretrione C (**62**) (Figure 16). Of all the isolated compounds, only compounds **57** and **62** were moderately active against *C. albicans* with MIC = 32 mg/L [46].

**Figure 17:**
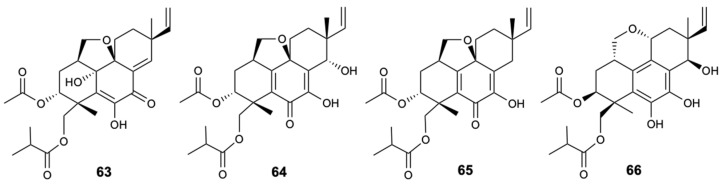
Structures of pimarane diterpenes (**63**–**66**).

Pimarane diterpenes are considered one of the secondary metabolites of great interest produced by the marine-derived fungus *Eutypella*, as they present a great structural variety and a wide range of pharmacological activities. Thus, *Eutypella* spp. D-1 was investigated in the Artic polar region, which subsequently led to the isolation of three new pimarane diterpene, eutypellenoids A–C (**63**–**65**), and a known compound, eutypenoid C (**66**) (Figure 17). All compounds were tested for their antifungal activity against *C. albicans*, *C. glabrata*, *C. parapsilosis*, *C. tropicalis*, *C. neoformans*, and *N. gypsea*, in which fluconazole, posaconazole, and voriconazole were used as positive controls. Only the compound **63** revealed activity against *Candida* spp., with MIC values of 8, 16, 8, and 32 mg/L, respectively [47].

**Figure 18:**
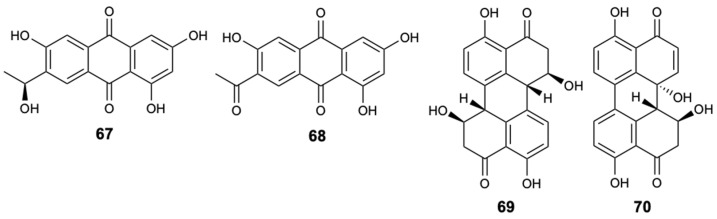
Structures of compounds **67**–**70**.

In the intertidal zones of the Yellow Sea in Qingdao, China, 141 strains of fungi were discovered from various marine plants, especially *Alternaria* sp. and *Fusarium* sp. Since these species are associated with metabolites with different biological activities, the selection of *F. equiseti* and *Alternaria* spp. from a screening of 31 identified strains led to the extraction of two new anthraquinone derivatives **67** and **68**, and two perylenequinones, stemphyperlenol (**69**) and alterpeylenol (**70**) (Figure 18). The compounds were tested for antifungal activity against a wide spectrum of six pathogenic fungi: *Alternaria alternata*, *Alternaria brassicicola*, *Phytophthora parasitica* var. *nicotianae*, *Diaporthe medusaea*, *A. niger*, and *Pestallozzia theae.* Compounds **67** and **68** demonstrated moderate action under *Pestallozzia theae* with MIC = 31.3 mg/L, while compound **69** inhibited not only the growth of *Pestallozzia theae*, but also of *Alternaria brassicicola* with MIC values of 7.81 and 125 mg/L, respectively, in which they were identical to the positive control, carbendazim [48].

**Figure 19:**
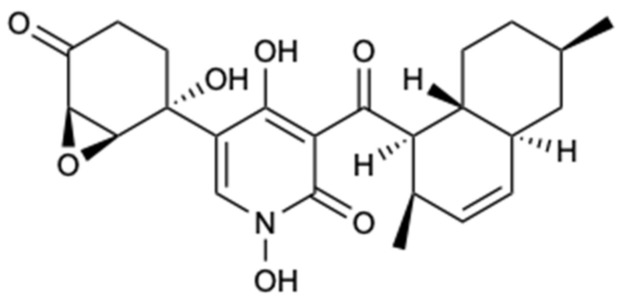
Structure of didymellamide A (**71**).

A new 4-hydroxy-2-pyridone alkaloids, didymellamide A (**71**) (Figure 19), was isolated from the marine fungus *Stagonosporopsis cucurbitacearum*. This compound was able to inhibit the growth of azole-resistant *C. albicans* J2-36 (MIC = 3.1 mg/L), azole-sensitive *C. albicans* J1-97 (MIC = 3.1 mg/L), *C. glabrata* J-92 (MIC = 3.1 mg/L), and *C. neoformans* Mpu-B (MIC = 1.6 mg/L). This study proposes that the didymellamide A hydroxamic acid fraction plays an important role in antifungal activity [49].

**Figure 20:**
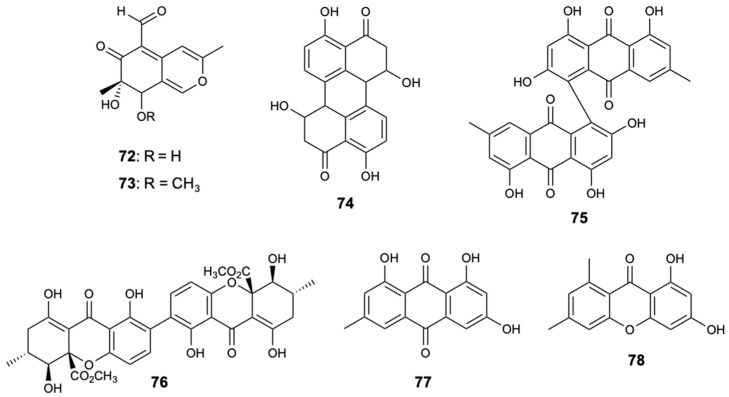
Structures of xanthones and structurally related compounds (**72**–**78**).

Two xanthones and structurally-related compounds, 7-epiaustdiol (**72**), 8-*O*-methylepiaustdiol (**73**), stemphyperylenol (**74**), skyrin (**75**), secalonic acid A (**76**), emodin (**77**), and norlichexanthone (**78**) (Figure 20), were isolated from the endophytic fungus *Talaromyces* spp. ZH-154, associated with mangrove plants, *Kandelia candel* (L.) Druce, collected in the South China Sea. All compounds were tested for antifungal inhibition against *C. albicans*, *A. niger*, and *F. oxysporum* f. sp. *cubense*, with nystatin used as a positive control. Compound **76** showed potent antifungal activity against all fungal strains tested. Notably, this compound showed inhibitory activity to *A. niger* with MIC = 6.25 mg/L, which is lower than nystatin (MIC = 25.0 mg/L) [50].

**Figure 21:**
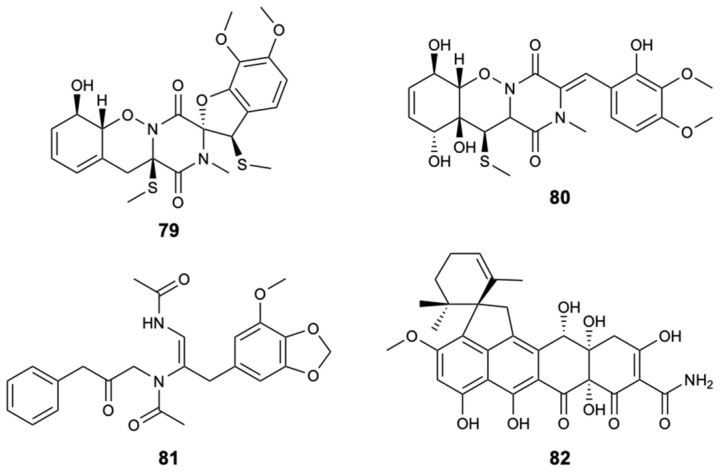
Structures of dithiodiketopiperazine derivatives (**79**–**82**).

The dithiodiketopiperazine derivatives represent a unique class of secondary fungal metabolites that usually contain two methythio groups or a disulfide bridge, in which, normally, the sulfur atom is attached to a-positions of the cyclic dipeptide. The chemical investigation of the fungal strain *Penicillium adametzioides* AS-53, a fungus isolated from an unidentified sponge, made it possible to extract two new dithiodiketopiperazine derivatives, peniciadametizines A,B (**79**,**80**), and two known substances, brasiliamide A (**81**) and viridicatumtoxin (**82**) (Figure 21). Compounds **79** and **80** were evaluated against four plant pathogenic fungi: *Alternaria brassicae*, *Colletotrichum gloeosporioides*, *F. graminearum*, and *Gaeumannomyces* spp. Both compounds demonstrated selective antifungal activity against *Alternaria brassicae* with MIC values of 4 and 32 mg/L, respectively, in comparison with AmB showing a MIC = 1.0 mg/L [51].

**Figure 22:**
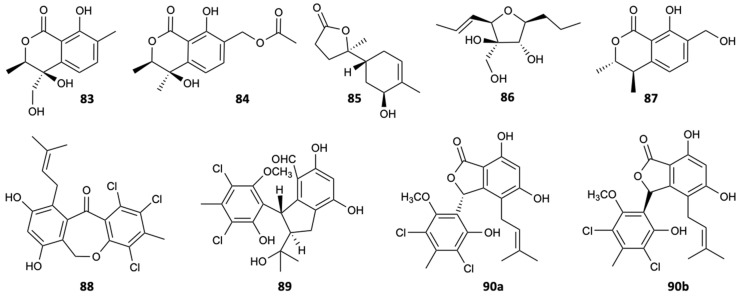
Structures of compounds **83**–**90a/90b**.

Fungi of the genus *Pestalotiopsis* are widely distributed around the world originating biologically active natural products. In the Xisha Islands of China, the fungal strain *Pestalotiopsis heterocornis* was discovered from the sponge *Phakellia fusca*, leading to the isolation of four new substances, pestaloisocoumarins A,B (**83**,**84**), isopolisin B (**85**), and pestalotiol A (**86**), which together with four known substances, gamahorin (**87**), pestalachloride B (**88**) and E (**89**), and a mixture of pestalalactone atropoisomers (**90a**/**90b**) (Figure 22), were submitted to antifungal activity evaluations against *C. albicans*, *C. parapsilosis*, and *C. neoformans*, being the antifungal AmB used as control with MIC ≤ 2.0 mg/L for the tested strains. Isocoumarins **83**, **84**, and **87** showed significantly weak activity with MIC = 100 mg/L against most of the fungi tested. Compounds **88**, **89**, and **90a**/**90b** were inactive at 100 mg/L [52].

### 2.4. Sea Cucumber-Derived Compounds

**Figure 23:**
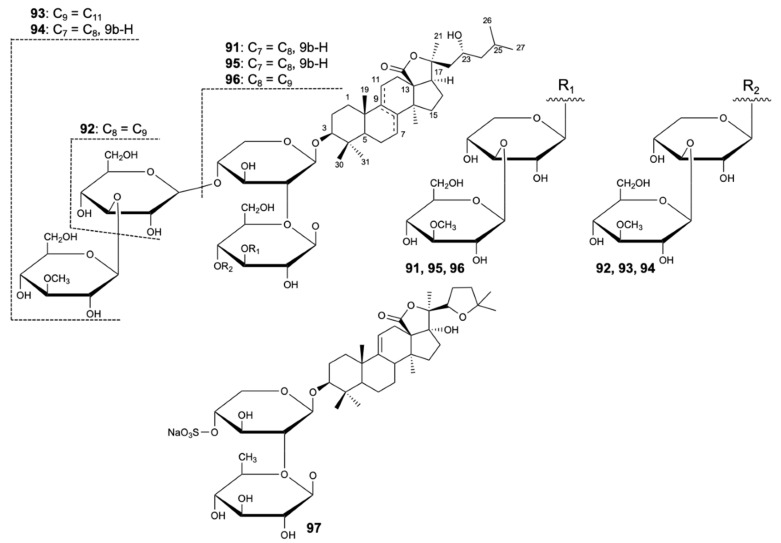
Structures of compounds **91**–**97**.

Four new compounds, variegatusides C–F (**91**–**94**), were isolated together with three known triterpene glycosides, variegatusides A,B (**95**,**96**), and holothurin B (**97**) (Figure 23) from the sea cucumber *Stichopus variegates* Semper. The compounds were tested for antifungal activity against *C. albicans*, *C. parapsilosis*, *C. tropicalis*, *C. pseudotropicalis*, *C. neoformans,* and *N. gypsea*, with ketoconazole, fluconazole, and itraconazole as positive controls. All the compounds studied showed selective activities except for compounds **92** and **93**, which revealed significant fungal growth inhibitory activities against the six species. Compound **92** exhibited activity with MIC_80_ values of 3.4, 3.4, 13.6, 3.4, 6.8, and 3.4 mg/L, respectively, while compound **93** showed MIC_80_ values of 25 mg/L for *C. albicans* and 12.5 mg/L for the remaining fungi [53].

**Figure 24:**
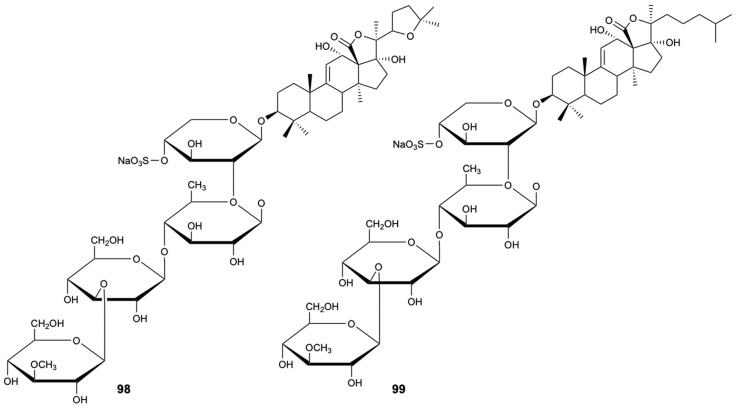
Structures of compounds **98** and **99**.

Two triterpenoid glycosides, holothurin A (**98**) and echinoside A (**99**) (Figure 24), were isolated from sea cucumber holothurian *Pearsonothuria graeffei* from the Red Sea, Egypt. The partial crude and purified extract from sea cucumber containing mainly these compounds was screened for antifungal activity against three clinical isolates of *C. albicans* (*Candida* 580, *Candida* 581, and *Candida* MEO47228), and the purified fraction showed good antifungal activity (24 h LC_50_ = 10 mg/L) [54].

Table 1 puts in evidence the chemical classes, sources, MIC values, and spectra of activities of the most remarkable compounds. It is interesting to highlight the synergic effects shown by a mixture formed by compounds **46** and **47**, compound **49** with AmB, and compounds **50**–**56** with fluconazole, and the effects against multi-resistant strains of *C. albicans.* Curiously, several secondary metabolites belong to the chemical class of polyketides, making it possible to establish a common structure–activity relationship (SAR), and marine sources have proved to be quite diverse. The most interesting compound appears to be Aurantoside I (**3**), as it revealed an excellent growth inhibition of all tested strains (*C. albicans*, *C. glabrata*, *C. parapsilosis*, *C. tropicalis*, and *F. solani*). Of particular interest is the activity shown against *F. solani*, since this strain is highly pathogenic and resistant to most antifungals available on the market. As all the studied compounds (aurantosides G–J, **1**–**4**) have a C18 polyene chain, the role of the sugar portion in the modulation of antifungal activity stands out.

## 3. Prospects for the Application of Marine Antifungal-Derived Compounds

The current therapeutic arsenal of antifungals leads to the rapid emergence of pathogenic fungi resistant and multi-resistant to antifungal agents, and consequently, it aggravates the treatment and/or prevention of fungal infections. Therefore, it is essential to look for new antifungal targets, as well as to explore new approaches. In this chapter, recent developments correlated with marine antifungal compounds will be presented as examples of new perspectives of application: new mechanisms of action, the combination of antifungal and non-antifungal agents, and the use of anti-virulence therapy and nanoparticles.

### 3.1. New Mechanisms of Action

**Figure 25:**
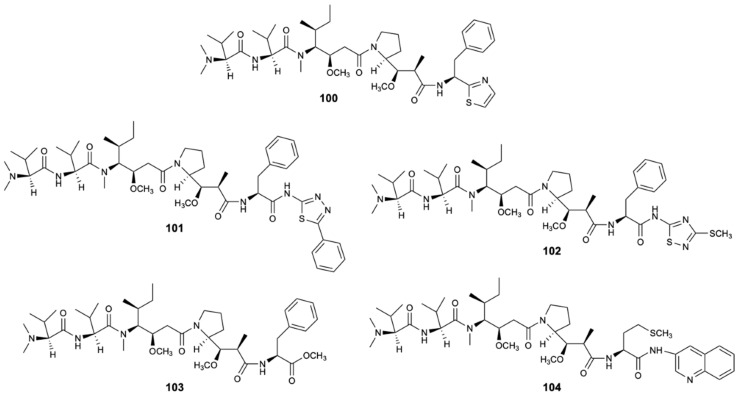
Structures of dolastatin 10 (**100**) and analogues **101**–**104**.

Dolastatin 10 (**100**) (Figure 25) is a natural product isolated from the Indian Ocean from the sea hare *Dolabella auricularia*. At the moment, studies have shown that dolastatin 10 and its derivatives are essentially derived from the sea hare’s diet of marine cyanobacteria, in particular *Symploca* sp. [55]. This linear peptide of three unique amino acid units proved to be able to inhibit the microtubules assembly and tubulin-dependent guanosine triphosphate binding by interfering in the formation of tubulin and thus interrupting cell division by mitosis and inducing apoptosis. Dolastatin 10 (**100**) and four other analog peptides (**101**–**104**) (Figure 25), obtained from **100** by structural modifications, showed specific fungicidal activity against ATCC strains and clinical isolates (including fluconazole-resistant strains) of *C. neoformans* with MIC_50_ = 0.195 mg/L and MIC_90_ = 0.39 mg/L [56,57].

### 3.2. Anti-Virulence Therapy

The growing number of pathogenic fungal strains that have become resistant to the conventionally used commercial antifungals constitute the main barrier in the treatment of these infections. Within this scenario, an innovative proposal for the effective control of the emergence and spread of fungal pathogens is the anti-virulence therapy. This strategy aims to selectively disarm the main virulence mechanisms of the pathogen. Among the advantages of using anti-virulence therapy, the following stand out: the development of new antifungal drugs that aim act at different targets and have new mechanisms of action; no interference with the host’s natural microbiota; and to exercise little selection pressure for drug resistance mutations, which has become a major drawback for *C. albicans* [58]. The filamentation process has been the main target of these studies, which were already validated at the genetic level, for the development of anti-virulence approaches in the treatment of candidiasis, even in immunocompromised patients. The search for specific inhibitors through the implementation of high-throughput screening, for subsequent in vitro and in vivo tests, has been the approach used in this pipeline for the development of new antifungal strategies.

**Figure 26:**
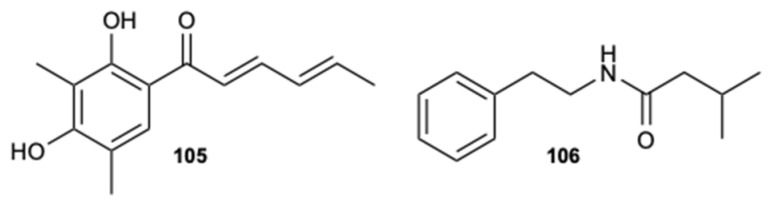
Structures of sorbicillin (**105**) and 3-methyl-*N*-(2′-phenelethyl)-butyrylamide (**106**).

Compounds sorbicillin (**105**) and 3-methyl-*N*-(2′-phenelethyl)-butyrylamide (**106**) (Figure 26), extracted from a crude extract of a deep-sea strain *Streptomyces olivaceus* SCSIO T05, demonstrated a potent ability to block the morphological transition (yeast-hyphae) reducing the formation of hyphae, the activity of adhesion to human cells (initial step for the formation of biofilm), and, consequently, the virulence of *C. albicans*. In addition, compound **106** showed a significant inhibitory effect in in vivo mouse oral mucosal models and in the expression levels of some specific promoters, such as HWP1, TEC1, ALS1, IFD6, and CSH1, which are associated with cell adhesion and the formation of hyphae. These preliminary results suggest that an anti-virulence strategy could potentially be used for the clinical treatment of candidiasis [59].

Another example of a marine natural product showing an antibiofilm effect is carboxymethyl chitosan. Chitin is the main structural component of the exoskeleton of marine invertebrates and the cell walls of fungi. Currently, the main commercial sources of this natural polysaccharide of great abundance and importance are shrimp and crab [60,61,62]. The partial deacetylation of chitin provides the formation of chitosan, which is a biocompatible, biodegradable, and non-toxic polymer widely studied for applications in the fields of biomedical, food, biotechnology, cosmetics, and pharmaceuticals. Chitosan has attracted a lot of attention due essentially to its diverse biological activities, such as antimicrobial and antioxidant activity. It is known that in fungi, chitosan acts at the level of plasma membranes and cell walls, chelating traces metals and inhibiting messenger RNA (mRNA) synthesis. Chitosan has proven to be highly active against *C. albicans*, and therefore, it can be considered as a potential anti-candidiasis agent. However, chitosan has some limitations, namely the solubility in water associated with its structural rigidity that makes it impossible to be applied in systems that require greater solubility and drug release rate. The low solubility of chitosan can be improved by hydrophilic modifications, such as the carboxymethylation that originates the carboxymethyl chitosan (CMC) derivative [61,63,64]. In vitro studies have shown that CMC has a strong inhibitory effect on *C. albicans, C. tropicalis*, *C. krusei*, *C. parapsilosis*, and *C. glabrata*, presenting a more prominent antifungal activity than chitosan. In addition, it has been demonstrated that CMC can inhibit the growth of biofilm, so it has been used in the area of medicine to prevent the formation of biofilms occurred during fungal infections on the surface of implanted devices [65]. However, in vivo studies are needed to prove the antifungal action of the CMC coating on silicone medical prostheses [66]. It has also found that CMC can be used as a gauze coating material, since the diameter of growth inhibition of the gauze coated with CMC was 0.30 cm, which is distinctly different compared to the zone of inhibition produced by the gauze coated with chitosan (0.12 cm) [61,64].

### 3.3. Combination of Antifungal and Non-Antifungal Agents

A strategy to overcome the emergence of resistant and multi-resistant fungi is the combination of antifungal agents present on the market. However, antifungal combination therapy is associated with harmful effects. To circumvent the high costs, the serious adverse effects, and the contradictory results of the reported synergistic or antagonistic activity associated with many antifungal combinations, the combination of antifungal with non-antifungal agents stands out. A common example of this approach is the combined use of fluconazole with other classes of non-antifungals, such as antibacterials, heat shock protein 90 inhibitors, and calcineurin inhibitors. In vitro studies revealed that many of the combinations demonstrated a synergistic effect and an increased susceptibility to strains resistant to existing antifungals, mainly in *C. albicans*. Examples of possible combination of antifungals and marine-derived compounds are presented.

#### 3.3.1. Combination with Efflux Pumps Inhibitors

**Figure 27:**
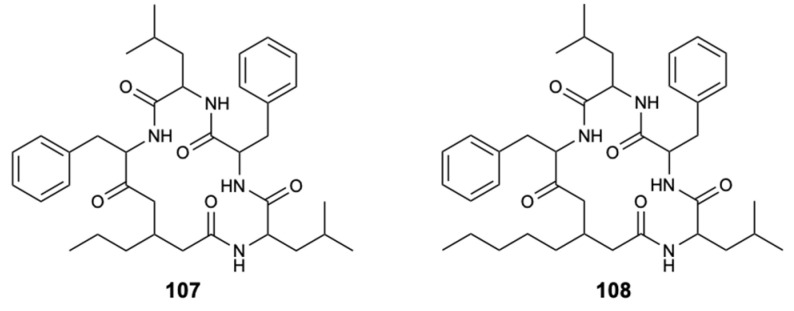
Structures of efflux pumps inhibitors: unnarmicin A (**107**) and unnarmicin C (**108**).

One of the main potential mechanisms of the observed synergistic activity is presumed to comprise the blocking of fungal plasma membrane efflux pumps. The active pumping of antifungals from the intracellular to the extracellular medium through efflux pumps leads to a subsequent reduction in the intracellular antifungal concentration resulting in resistance. Thus, a viable strategy for overcoming antifungal resistance is to inhibit efflux pumps [67,68,69]. Unnarmicin A (**107**) and unnarmicin C (**108**) (Figure 27) are candidate inhibitors of the resistance mechanism, namely efflux pumps. These natural compounds (also from marine sources) showed a synergistic effect with fluconazole, which potentiated the antifungal activity against azole-resistant *C. albicans*, and thus can be considered as potential adjuvants in antifungal therapy. Thus, these results lead to the conclusion that marine inhibitors of efflux pumps are promising compounds and can be of help in reversing the problem of antifungal resistance [69,70].

#### 3.3.2. Combination with Compounds that Induce the Reactive Oxygen Species Formation

*Candida* spp. are the main fungal pathogens responsible for infections in both the mucosa and deep tissues. The pathogenicity of these yeasts is attributed to certain virulence factors, and recent studies suggest that most invasive infections produced by *Candida* spp. are associated with the formation and growth of biofilms in host tissues and medical devices. Furthermore, biofilms are identified to have more antifungal resistance compared to planktonic cells, and therefore, they play an important role in the perpetuation of infections [71,72]. Currently, there are few antifungals, such as miconazole, AmB, and echinocandins, which are also effective against fungal biofilms. These antifungals, in addition to their demonstrated and main mechanism of action, also revealed ability to inhibit biofilms by stimulating reactive oxygen species (ROS) production in both planktonic and biofilm cells. Thus, a promising approach to control biofilm formation is the induction of ROS. Miconazole is capable of affecting *C. albicans* biofilms; nonetheless, for high concentrations, the presence of persistent cells resistant to the action of miconazole was observed due to the activity of superoxide dismutases with a consequent reduction of ROS [73,74,75]. Considering polyenes, AmB and AmB lipid formulations showed to be effective against mature *C. albicans* biofilms, and they had high activity in a model of central venous catheter in rabbits and mice. However, as seen with miconazole, the treatment of biofilms requires higher concentrations of AmB [75,76,77]. Finally, echinocandins (anidulafungin, caspofungin, and micafungin) also showed activity against *Candida* biofilms; similar activity for biofilms and planktonic cells was reported, with caspofungin being the echinocandin most effective. However, so far, there are still no reports on the induction of ROS in fungal biofilms by this class [75,77,78]. Recently, new metabolites isolated from several sources showed as a specific or additional mechanism of action the accumulation of ROS and, subsequently, the induction of apoptosis in fungal pathogens. One example is the marine polyunsaturated fatty acids, which is a class newly identified with inhibitory action against biofilms formation from *C. albicans* and *C. dubliniensis*. In particular, stearidonic acid (18:4 n-3), eicosapentaenoic acid (20:5 n-3), and docosapentaenoic acid (22:5 n-3) were able to inhibit the mitochondrial activity of biofilms and affect the cell morphology of biofilms from both strains [75,79]. Fluconazole is an antifungal that is widely used in the treatment and prophylaxis of fungal infections that shows reduced ability to induce ROS; its combination with molecules capable of stimulating the ROS production could result in a synergistic effect. Although the induction of ROS and, eventually, the occurrence of apoptosis in fungal cells can be considered a very promising approach for the treatment and/or prevention of *Candida* biofilms, further studies are needed to evaluate the relationship between the mode of action and the presence of ROS [75].

### 3.4. Nanoparticles

Some current antifungal agents present major obstacles, such as hydrophobic character, toxicity, pharmacological interactions, low aqueous solubility, and low oral bioavailability, which limit their clinical benefits. Thus, the development of drug delivery systems is a promising strategy for improving the performance and safety profile of antifungals, maintaining or increasing their therapeutic efficacy, and overcoming many of these associated limitations. Of the many drug delivery systems currently under investigation, nanoparticles have emerged as an innovative way that is capable of minimizing undesirable adverse effects and overcoming many of the unfavorable properties of antifungals. Due to a wide range of advantageous and captivating characteristics, involving reduced size, variability, improved stability, multifunctionality, biocompatibility, directing target tissues, and the possibility of increasing the penetration of antifungal agents through the skin, nanocarriers were hypothesized to assist in the treatment of invasive fungal infections. Liposomes, solid lipid nanoparticles, and nanostructured lipid carriers are some of the most studied lipid-based nanocarriers for the delivery of antifungals on the treatment of invasive mycoses in numerous clinical trials. A visible example of the application of nanoparticles as a tool for drug delivery systems is with AmB, where the use of formulations based on nanoparticles, such as AmB lipid complex, AmB colloidal dispersion, and liposomal AmB, demonstrated minimal or no toxicity, maintaining its broad-spectrum antifungal activity. However, the study of nanoformulations in the antifungal area has been very gradual, unlike in the field of cancer diagnosis and therapy, with AmB being almost the only commercially available antifungal in different nanoparticle formulations. This situation is mainly due to the divestment of the pharmaceutical industry and the lack of economic incentives, as the production of nanoparticles involves a high cost, due to the limitations of the nanoparticles themselves, such as low physical stability and pharmacokinetic/biodistribution profiles, and the poor correlation between in vitro and in vivo trials [80,81]. Studies revealed that conjugated systems of antifungals, such as azoles and AmB, and metallic nanoparticles, mainly prepared from metals such as Ag, Pt, Au, and Pd, have a synergistic action. This is the case of the combination of silver nanoparticles (Ag-NPs) with fluconazole and itraconazole. The use of a marine mangrove extract (*Rhizophora mucronata*) and silver nitrate allowed synthesizing nanoparticles that revealed an inhibitory and fungicidal effect, and when conjugated with fluconazole and itraconazole, it significantly increased the activity against *C. albicans* and *A. fumigatus*. Thus, it can be suggested that these formulations have a synergistic effect when associated with azoles [82]. Magnesium oxide nanoparticles (MgO-NPs), prepared from a marine brown algae *Sargassum wighitii*, were also found as effective agents against pathogenic fungi. In comparison with fluconazole (positive control), nanoparticles demonstrated a potent inhibition against *A. fumigatus*, *A. niger*, and *F. solani*, being *A. fumigatus* less susceptible [83]. Given their biocompatibility and stability in adverse conditions, these metal oxide nanoparticles have been applied for the relief of heart burns and the regeneration of bones and used as antitumor and antibacterial agents. Since seaweeds/marine algae are easily accessible and of great associated effectiveness, their use in the synthesis of nanoparticles has become an essential and predominant recent step. These results suggest that the use of marine-derived associated-nanoparticles systems may be a promising approach for the treatment of fungal infections.

## 4. Conclusions

This review focuses on new bioactive compounds from marine sources and new perspectives for their antifungal application. Currently, there are many challenges in the treatment and prevention of fungal infections due to the increase in the number of cases and the emergence of resistance (primary, secondary, and biofilm formation) to the few antifungal agents available in clinical practice. In addition, the unfavorable characteristics of current antifungals have led to the need to seek new treatment options.

Given that the marine environment represents a huge and still unexplored source of secondary metabolites with a wide structural variety and a wide range of pharmacological activities, natural products with potential in vitro antifungal activity isolated from numerous marine sources were addressed. In this review, it is worth highlighting a combination formed by compounds **46** and **47**, compound **49** with AmB, and compounds **50**–**56** with fluconazole, which exhibited a synergistic effect with greater antifungal action than any of the isolated compounds. Almost all of the compounds mentioned inhibited the growth of a diversity of fungal species, with special attention to compounds **1**, **3**, **5**, **9**, **19**, **37**, **39**, **40**, **42**, **49**, **52**, **53**, **55**, **56**, **63**, **71**, **76**, and **92** that showed potent activity against strains of *C. albicans*. Although several promising marine natural products to antifungal agents have been found, their development has been progressive, as this is a very time-consuming process, and the amount of products derived in particular from sponges is limited due to cost and biodiversity, as well as the difficulty in synthesizing them chemically.

Understanding the pathogenic processes of fungi contributed to the discovery of new targets and, consequently, new inhibitors, making new targets a good alternative to develop new antifungals. However, this strategy remains challenging, because fungal cells share a high physiological similarity with human cells and, thus, several targets involved in the cell membrane and the biosynthesis of proteins or deoxyribonucleic acid (DNA) are not specific to fungi. The inhibition of microtubule assembly is a new potential target and, consequently, it is a new mechanism of action used to combat the proliferation of fungi and infections. The results obtained suggest that the application of its inhibitor, such as dolastatin 10 (**100**), will be able to block the growth of even strains of resistant pathogenic fungi. However, it is necessary to continue to discover, evaluate, and optimize more promising inhibitors, although many antifungal targets with therapeutic potential have already been found and identified in clinical trials. Currently, studies focused on increasing the activity of antifungals, especially fluconazole, have revealed a combination therapy of antifungal and non-antifungal agents as an option to deal with the problems associated with the treatment of invasive fungal infections and antifungal resistance, and it may become a way to solve the limited therapeutic arsenal of antifungals. Although the mechanism of action of commercial antifungals has already been extensively investigated, some of these when combined with marine compounds that inhibit efflux pumps exhibit a synergistic effect. This situation proves to be another interesting strategy for combating antifungal resistance. On the other hand, the direction of the oxidative defense system of pathogenic fungi can also be a powerful tool for increasing the activity of antifungals currently available in therapy and allowing dosage reduction, given that ROS induction is correlated with fungicidal activity. In addition, ROS has been shown to be a promising application to overcome the formation of biofilms in the development of invasive fungal infections. Therefore, the discovery of compounds that can act by this mechanism, associated or not with the existing antifungals, can help in the fight against fungal infections related or not with biofilms. Chitosan is a polymer of great interest in antifungal therapy as a possible anti-candidiasis agent, given the potent activity revealed on *C. albicans*. A remarkable solution that improves the solubility in water of chitosan is derivatization by carboxymethylation as a hydrophilic modification to produce CMC. This derivative presents greater antifungal activity in comparison to chitosan, a broad spectrum of action in *Candida* spp., and the ability to interfere in the formation of biofilms. Although there are some effective antifungal agents, their therapeutic benefits are limited by high toxicity or undesirable physicochemical properties. The use of nanoparticle formulations such as antifungal delivery systems can overcome these limitations. Another innovative strategy for the effective control of the emergence and spread of fungal pathogens is the anti-virulence therapy, which aims to disarm the virulence mechanisms of the pathogen, with fungi filamentation being the main target of study. Preliminary results suggest that this application could potentially be used to treat certain forms of candidiasis; however, more studies must be carried out to prove their real effectiveness.

## Figures and Tables

**Table 1:** The general characteristic of the marine natural products with antifungal activity.

Compound	Chemical Class	Source	Activity	References
Aurantoside G (**1**)	Peptide	Sponge *Theonella swinhoei*	*C. albicans*, *C. glabrata*, *C. parapsilosis*, and *C. tropicalis* (MIC_90_ 8, 8, 4, and 4 mg/L)	[30]
Aurantoside I (**3**)	Peptide	Sponge *Theonella swinhoei*	*C. albicans*, *C. glabrata*, *C. parapsilosis*, *C. tropicalis*, and *F. solani* (MIC_90_ 0.5, 0.125, 0.5, 0.5, and 2 mg/L)	[30]
Aurantoside K (**5**)	Peptide	Sponge *Meophlus* sp.	AmB-R and WT *C. albicans* (MIC 31.25 and 1.95 mg/L); *C. neoformans*, *A. niger*, *Rhizopus sporangia*, *Penicillium* sp., and *Sordaria* sp. (Ø inhibition 14, 28, 21, 31, and 29 mm)	[31]
Woodylide A (**6**)	Polyketide	Sponge *Plakortis simplex*	*C. neoformans* (IC_50_ 3.67 mg/L) *C. albicans*, *N. gypsea*, and *T. rubrum* (MIC 32 mg/L)	[32]
Woodylide C (**8**)	Polyketide	Sponge *Plakortis simplex*	*C. neoformans* (IC_50_ 10.85 mg/L); *N. gypsea* and *T. rubrum* (MIC 32 mg/L)	[32]
Theonellamide G (**9**)	Peptide	Sponge *Theonella swinhoei*	AmB-R and WT *C. albicans* (IC_50_ 4.49 and 2.0 µM)	[33]
15-Formamido-kalihinene (**18**)	Terpene	Sponge *Acanthella cavernosa*	*N. gypsea* and *T. rubrum* (MIC 8 and 32 mg/L)	[34]
10-Formamido-kalihinene (**19**)	Terpene	Sponge *Acanthella cavernosa*	*C. albicans*, *C. neoformans*, *N. gypsea*, and *T. rubrum* (MIC 8, 8, 8, and 4 mg/L)	[34]
Aaptamine (**24**)	Alkaloid	Sponge *Aaptos aaptos*	*C. parapsilosis* (MIC 32 mg/L)	[35]
Aaptamine (**25**)	Alkaloid	Sponge *Aaptos aaptos*	*N. gypsea* (MIC 64 mg/L)	[35]
Aaptamine (**26**)	Alkaloid	Sponge *Aaptos aaptos*	*N. gypsea* (MIC 64 mg/L)	[35]
Aaptamine (**27**)	Alkaloid	Sponge *Aaptos aaptos*	*N. gypsea* (MIC 64 mg/L)	[35]
Aaptamine (**28**)	Alkaloid	Sponge *Aaptos aaptos*	*C. albicans*, *C. parapsilosis*, *C. neoformans*, *N. gypsea*, and *T. rubrum* (MIC 32, 64, 32, 16, and 4 mg/L)	[35]
Aaptamine (**29**)	Alkaloid	Sponge *Aaptos aaptos*	*C. neoformans*, *N. gypsea*, and *T. rubrum* (MIC 64, 32, and 8 mg/L)	[35]
Poecillastroside D (**33**)	Steroid	Sponge *Poecillastra compressa*	*A. fumigatus* (MIC_90_ 6 mg/L)	[36]
Poecillastroside E (**34**)	Steroid	Sponge *Poecillastra compressa*	*A. fumigatus* (MIC_90_ 24 mg/L)	[36]
Haliscosamine (**37**)	Polyketide	Sponge *Haliclona viscosa*	*C. albicans*, *C. tropicalis*, and *C. neoformans* (MIC_90_ 0.4–0.8, 0.4–0.8, and 0.2–0.4 mg/L)	[38]
Epi-ilimaquinone (**38**)	Polyketide	Sponge *Hippospongia* sp.	AmB-R *C. albicans* (MIC 125 mg/L)	[39]
Isoikarugamycin (**39**)	Polyketide	Bacteria *Streptomyces zhaozhouensis*	*C. albicans* and *A. fumigatus* (MIC 2–4 and 4–8 mg/L)	[40]
28-*N*-methylikaguramycin (**40**)	Polyketide	Bacteria *Streptomyces zhaozhouensis*	*C. albicans* and *A. fumigatus* (MIC 4 and 4–8 mg/L)	[40]
Ikarugamycin (**42**)	Polyketide	Bacteria *Streptomyces zhaozhouensis*	*C. albicans* and *A. fumigatus* (MIC 4 and 4–8 mg/L)	[40]
Lobocyclamide A (**46**)	Peptide	Cyanobacterium *Lyngbya confervoides*	*C. albicans* (MIC 91 mg/L) Synergism with mixture of **46** and **47** against *C. albicans* (MIC 10–30 mg/L)	[42]
Lobocyclamide B (**47**)	Peptide	Cyanobacterium *Lyngbya confervoides*	*C. albicans* (MIC 30-100 mg/L) Synergism with mixture of **46** and **47** against *C. albicans* (MIC 10–30 mg/L)	[42]
Forazoline A (**49**)	Polyketide	Bacteria *Actinomadura* spp.	*C. albicans* (MIC < 16 mg/L); Synergism with AmB	[43]
PF1163A (**52**)	Polyketide	Fungus *Penicillium meleagrinum* var*. viridiflavum*	Azole-resistant *C. albicans* (MIC 1 mg/L) Synergism with fluconazole against the azole-resistant *C. albicans*	[45]
PF1163B (**53**)	Polyketide	Fungus *Penicillium meleagrinum* var*. viridiflavum*	Azole-resistant *C. albicans* (MIC 2 mg/L) Synergism with fluconazole against the azole-resistant *C. albicans*	[45]
PF1163H (**55**)	Polyketide	Fungus *Penicillium meleagrinum* var*. viridiflavum*	Azole-resistant *C. albicans* (MIC 16 mg/L) Synergism with fluconazole against the azole-resistant *C. albicans*	[45]
PF1163F (**56**)	Polyketide	Fungus *Penicillium meleagrinum* var*. viridiflavum*	Azole-resistant *C. albicans* (MIC 8 mg/L) Synergism with fluconazole against the azole-resistant *C. albicans*	[45]
Terretrione D (**57**)	Alkaloid	Fungus *Penicillium* sp. CYE-87	*C. albicans* (Ø inhibition 17 mm and MIC 32 mg/L)	[46]
Terretrione C (**62**)	Alkaloid	Fungus *Penicillium* sp. CYE-87	*C. albicans* (Ø inhibition 19 mm and MIC 32 mg/L)	[46]
Eutypellenoid A (**63**)	Terpene	Fungus *Eutypella* sp. D-1	*C. albicans*, *C. glabrata*, *C. parapsilosis* and *C. tropicalis* (MIC 8, 16, 8, and 32 mg/L)	[47]
Anthraquinone (**67**)	Polyketide	Fungus *Fusarium equiseti*	*Pestallozzia theae* (MIC 31.3 mg/L)	[48]
Anthraquinone (**68**)	Polyketide	Fungus *Fusarium equiseti*	*Pestallozzia theae* (MIC 31.3 mg/L)	[48]
Stemphyperlenol (**69**)	Polyketide	Fungus *Alternaria* sp.	*Pestallozzia theae* and *Alternaria brassicicola* (MIC 7.81 and 125 mg/L)	[48]
Didymellamide A (**71**)	Alkaloid	Fungus *Stagonosporopsis cucurbitacearum*	Azole-resistant *C. albicans* J2-36, azole-sensitive *C. albicans* J1-97*, C. glabrata* J-92, and *C. neoformans* Mpu-B (MIC 3.1, 3.1, 3.1, and 1.6 mg/L)	[49]
Secalonic acid A (**76**)	Polyketide	Fungus *Talaromyces* sp. ZH-154	*C. albicans*, *A. niger* and *F. oxysporum* f. sp. *cubense* (MIC 6.25, 6.25, and 12.5 mg/L)	[50]
Peniciadametizine A (**79**)	Polyketide	Fungus *Penicillium adametzioides* AS-53	*Alternaria brassicae* (MIC 4.0 mg/L)	[51]
Peniciadametizine B (**80**)	Polyketide	Fungus *Penicillium adametzioides* AS-53	*Alternaria brassicae* (MIC 32 mg/L)	[51]
Pestaloisocoumarin A (**83**)	Lactone	Fungus *Pestalotiopsis heterocornis*	*C. albicans*, *C. parapsilosis*, and *C. neoformans* (MIC 100 mg/L)	[52]
Pestaloisocoumarin B (**84**)	Lactone	Fungus *Pestalotiopsis heterocornis*	*C. neoformans* (MIC 100 mg/L)	[52]
Gamahorin (**87**)	Lactone	Fungus *Pestalotiopsis heterocornis*	*C. parapsilosis* and *C. neoformans* (MIC 100 mg/L)	[52]
Variegatuside D (**92**)	Terpene	Sea cucumber *Stichopus variegates*	*C. albicans*, *C. parapsilosis*, *C. tropicalis*, *C. pseudotropicalis*, *C. neoformans*, and *N. gypsea* (MIC_80_ 3.4, 3.4, 13.6, 3.4, 6.8, and 3.4 mg/L)	[53]
Variegatuside E (**93**)	Terpene	Sea cucumber *Stichopus variegates*	*C. albicans*, *C. parapsilosis*, *C. tropicalis*, *C. pseudotropicalis*, *C. neoformans*, and *N. gypsea* (MIC_80_ 25, 12.5, 12.5, 12.5, 12.5, and 12.5 mg/L)	[53]
Holothurin A (**98**)	Terpene	Sea cucumber *Pearsontrhuria graeffei*	*C. albicans* (24 h LC_50_ = 10 mg/L)	[54]
Echinoside A (**99**)	Terpene	Sea cucumber *Pearsontrhuria graeffei*	*C. albicans* (24 h LC_50_ = 10 mg/L)	[54]

MIC: Minimum inhibitory concentration; MIC_80_: Minimum concentration that inhibits 80% of the tested strains; MIC_90_: Minimum concentration that inhibits 90% of the tested strains; Ø inhibition: Diameter of inhibition; IC_50_: Half maximal inhibitory concentration; LC_50_: Concentration that causes the death of 50%. *C. albicans*: *Candida albicans*; *C. glabrata*: *Candida glabrata*; *C. parapsilosis*: *Candida parapsilosis*; *C. tropicalis*: *Candida tropicalis*; *C. pseudotropicalis*: *Candida pseudotropicalis*; AmB-R: Amphotericin B-resistant; WT: Wild-type; *A. fumigatus: Aspergillus fumigatus*; *A. niger*: *Aspergillus niger*; *C. neoformans*: *Cryptococcus neoformans*; *N. gypsea*: *Nannizzia gypsea*; *F. solani*: *Fusarium solani*; *F. oxysporum*: *Fusarium oxysporum*; *T. rubrum*: *Trichophyton rubrum.*
